# Antitumor and Immunomodulatory Properties of the Bulgarian Endemic Plant *Betonica bulgarica* Degen et Neič. (Lamiaceae)

**DOI:** 10.3390/plants11131689

**Published:** 2022-06-26

**Authors:** Tsvetelina Mladenova, Tsvetelina Batsalova, Balik Dzhambazov, Rumen Mladenov, Ivanka Teneva, Plamen Stoyanov, Anelia Bivolarska

**Affiliations:** 1 Faculty of Biology, University of Plovdiv “Paisii Hilendarski”, 24 Tsar Assen Str., 4000 Plovdiv, Bulgaria; cmladenova@uni-plovdiv.bg (T.M.); tsvetelina@uni-plovdiv.bg (T.B.); balik@uni-plovdiv.bg (B.D.); rummlad@uni-plovdiv.bg (R.M.); teneva@uni-plovdiv.bg (I.T.); pstoyanov@uni-plovdiv.bg (P.S.); 2 Department of Medical Biochemistry, Faculty of Pharmacy, Medical University of Plovdiv, 15A Vasil Aprilov Blvd., 4002 Plovdiv, Bulgaria

**Keywords:** *Betonica bulgarica*, inflorescence extract, cytotoxicity, antitumor activity, immunomodulatory properties

## Abstract

**Background:** Extracts obtained from different *Betonica* species have been shown to possess important biological properties. The present study aimed to investigate the cytotoxicity, antitumor and immunomodulatory potential of the endemic plant *Betonica bulgarica* (Lamiaceae) and thus, reveal new aspects of its biological activity. **Methods:** Methanolic extract obtained from inflorescences was analyzed for cytotoxicity against mammalian cell lines. The antitumor potential of the sample was determined using human cervical and lung adenocarcinoma cells (HeLa and A549). Programmed cell death-inducing effects against HeLa cells and peripheral blood lymphocytes, as well as immunomodulatory properties of the extract were determined by flow cytometry analysis. **Results:** The research results demonstrated that the extract has significant inhibitory potential against HeLa cells (mean IC_50_ value 119.2 μg/mL). The sample selectively induced apoptotic death in tumor cells. Cytotoxic effects towards mouse cell lines were detected following treatment with high concentrations of *Betonica bulgarica* extract (200 and 250 μg/mL). Twenty-four-hour ex vivo incubation of peripheral blood leucocytes in growth medium containing plant extract induced prominent effects in distinct immune cell populations. They included elevated levels of CD25^+^ and CD56^+^ T cells’ lymphocytes, particularly CD4^+^CD25^+^ and CD8^+^CD56^+^ cells. **Conclusions:** The present study demonstrates that *Betonica bulgarica* inflorescence extract possesses potential beneficial antitumor and immunomodulatory activity and could serve as a source of bioactive compounds with biomedical application.

## 1. Introduction

*Betonica bulgarica* Degen et Neič. (synonym *Stachys bulgarica* Hayek) is an endemic plant that belongs to the Lamiaceae family [1]. It grows on carbonate, grey and brown forest soils in the Central and Eastern Balkan range and in the Thracian plain [1,2]. There is a significant amount of information about the general ecological, anatomical and morphological features (including palinomorphological traits) of this endemic species growing in Bulgaria [3,4]. Analyses of chemical composition, mineral and flavonoid content of several *Betonica bulgarica* populations have been reported [4,5,6]. Levels of trace elements and phytochemical composition of essential oils obtained from leaves, flowers and stems of the plant have also been determined [3].

Although many authors include *Betonica* within the *Stachys* taxonomic rank, morphological features and chemotaxonomic markers indicate that they form a distinct genus [7,8,9]. Representatives of both genera have been used in folk and homeopathic medicine due to their various biological activities (antioxidant, anti-inflammatory, antimicrobial, cytotoxic, antitumor, hypotensive, anti-nephritic, anxiolytic, etc.) [10,11]. Extracts obtained from *Betonica officinalis* which shares general taxonomic characteristics with *Betonica bulgarica* have been shown to have antioxidant [12], anti-inflammatory [13,14], antiproliferative and antitumor properties [15], as well as antidiabetic potential based on defined enzyme-inhibitory effects [16]. Recently, it has been found that methanolic extracts from the endemic *Betonica bulgarica* harvested from different locations in Bulgaria show moderate antibacterial activity [17] and also demonstrated good antioxidant properties that correlated with flavonoid and total phenolic content of the studied samples [18]. The significant amount of sesquiterpenes, flavonoids (mainly rutin, quercetin, hispidulin) and other phenolic compounds [3,18] suggest that extracts and essential oils derived from *Betonica bulgarica* could have broader biological activity exceeding the determined antibacterial and antioxidant properties and thus, could induce beneficial effects on human health. To date, the cytotoxicity profile and antitumor properties of the plant have not been investigated. Therefore, the present study aimed to evaluate the antitumor and immunomodulatory properties of *Betonica bulgarica* extracts.

## 2. Results

### 2.1. Antitumor Activity and In Vitro Cytotoxicity of Betonica Bulgarica

The cytotoxic activity of *Betonica bulgarica* was investigated in vitro using human and murine cell lines. The experiments allowed determination of antitumor potential because two of the examined cell lines were with adenocarcinoma origin (HeLa and A549) and one of the murine cell lines (RAW264.7) was tumor-derived. FL cell line derived from normal human amnion and mouse embryonic NIH/3T3 fibroblasts served as a control in order to compare cytotoxic effects between tumor and nontumor cells. The experiments included evaluation of extracts derived from different aerial parts of the plant—leaves, stems and inflorescences. As shown in Table 1, the inflorescence extract induced the most prominent inhibition of the human tumor cell line HeLa but did not affect to the same extent the nontumor mouse and human cells. On the contrary, the leaf extract induced strong inhibition of FL cells and the stem extract showed highest cytotoxicity against mouse nontumor cells.

**Figure 1:**
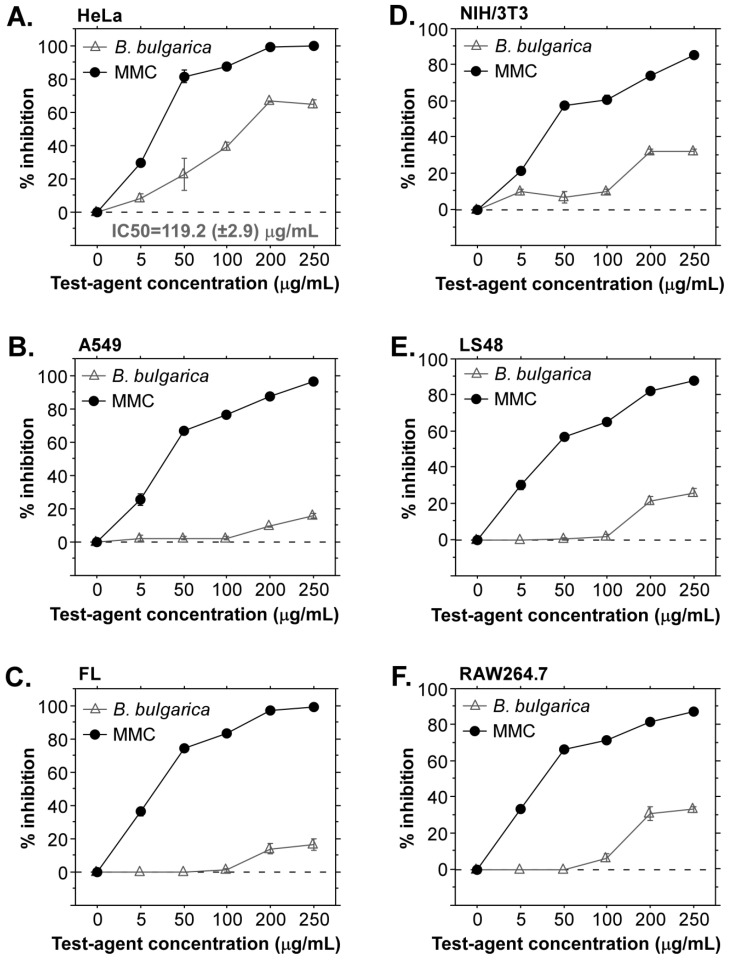
Cytotoxic effects of *Betonica bulgarica* extract against different human and murine cells. Human and mouse cells were cultured for 24 h in growth medium containing different concentration of *Betonica bulgarica* extract. After that, MTT assays were applied to evaluate the inhibitory effects on cell viability and metabolic activity. Results are displayed as ±standard error of the mean (SEM) and compared to the effect of mitomycin C, which served as a positive control for the assays. All samples were analyzed in triplicates. MTT assay results for HeLa (**A**), A549 (**B**) and FL (**C**) cells. Inhibition levels determined in the murine cell lines NIH/3T3 (**D**), LS48 (**E**) and RAW264.7 (**F**) after treatment with *Betonica bulgarica* extract.

These results depend on the complex composition of the samples and indicate antitumor potential of the inflorescence extract. Therefore, further biological activity evaluations included only the inflorescence extract. Figure 1 illustrates the results from the in vitro assays with this sample. They indicated a significant antitumor activity of *Betonica bulgarica*, which was specific for cervical adenocarcinoma cells (Figure 1A). The detected level of A549 cells’ inhibition was similar to the results with nontumor FL cells. Lung adenocarcinoma A549 cells did not show high sensitivity to the extract sample, which could be due to specific resistance of the cell line to the compounds with antitumor activity in the extract. The highest concentrations of the extract (200 and 250 μg/mL) induced mild cytotoxicity in the range of 10 to 20% inhibition (Figure 1B,C). These results stimulate future experiments with a panel of different tumor cell types in order to identify additional specific effects and broader antitumor activity.

Three mouse cell lines were included in the evaluations with the aim to clarify if the extract from *Betonica bulgarica* has a common cytotoxic or antitumor potential and affects cells from different mammalian species. Figure 1D–F demonstrate that the studied different murine cell types (embryonic fibroblasts, fibroblast-like synoviocytes, monocytic/macrophage cells) show a similar level of inhibition following 24 h incubation in culture medium containing *Betonica bulgarica* extract. Exposure to higher concentrations (200 and 250 μg/mL) resulted in 20 to 40% inhibition of cellular viability and metabolic activity. In this regard, LS48 cells displayed lower reduction of metabolic activity compared to RAW264.7 and NIH/3T3 cell cultures, which could be due to the intensive proliferative activity characteristic for this fibroblast-like cell line and lack of murine-specific anti-proliferative effect of the extract from *Betonica bulgarica*.

### 2.2. Phenolic Content of Betonica bulgarica Extracts

Phenolic compounds considerably contribute to the biological properties of plant-derived extracts. Therefore, the total phenolic content of Betonica bulgarica extracts obtained from leaves, stems and inflorescences was analyzed. Table 2 indicates that the leaf extract contained the highest level of total phenolic compounds. HPLC analyses pointed that the major phenolic substances in Betonica bulgarica methanolic extracts were chlorogenic and caffeic acid (Table 2). Although total phenolic content of the inflorescence extract was lower compared to the leaf extract, similar levels of chlorogenic and caffeic acid were determined. These results together with the in vitro cytotoxicity evaluations motivated the selection of inflorescence extract for further biological activity analyses.

### 2.3. Tumor Cell-Specific Proapoptotic Potential of Betonica bulgarica

**Figure 2:**
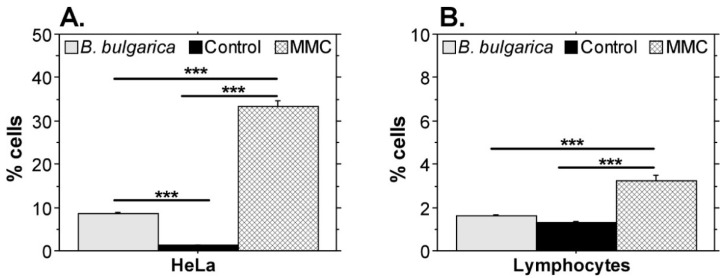
Levels of apoptotic cell populations. (**A**) % apoptotic HeLa cells, (**B**) Annexin V-positive peripheral blood lymphocytes. HeLa cells and peripheral blood leucocytes treated for 24 h with *Betonica bulgarica* extract or mitomycin C were stained with Annexin V-FITC to estimate the levels of apoptotic cells. Control cells were cultured in standard growth medium for the same period. Data are shown as ±SEM. The graphs represent pooled data from two independent experiments. All samples were analyzed in triplicates. ***, *p* < 0.001, determined by Fisher’s PLSD test. Significant differences between the control and *B. bulgarica*-treated group were confirmed by the Mann–Whitney U-test. MMC—mitomycin C; Control—cells cultured for 24 h in complete DMEM without *Betonica bulgarica* extract.

The results shown in Figure 2 demonstrate that *Betonica bulgarica* extract treatment significantly increased the number of apoptotic HeLa cells. Such effect was not detected in the sample with extract-treated leucocytes compared to the cells cultured in standard growth medium. All main leucocyte populations (granulocytes, monocytes, lymphocytes), which were distinguished by size and cellular granularity based on the forward and side scatter detectors’ evaluation were included in the analysis. They showed a similar trend—lack of proapoptotic effect in the extract-treated sample. This result is represented by the data for lymphocytes in Figure 2B and convincingly demonstrates tumor cell-specific induction of apoptosis following treatment with *Betonica bulgarica* extract.

### 2.4. Betonica bulgarica Extract Upregulated Particular Immune Cell Phenotypes

**Figure 3:**
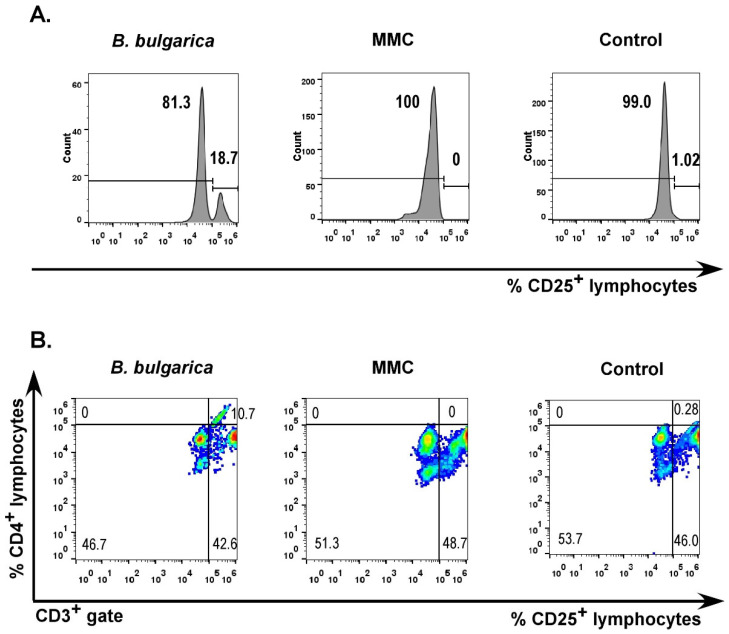
CD25^+^ immune cell populations. Peripheral blood leucocytes treated for 24 h with *Betonica bulgarica* extract or mitomycin C. Control cells were cultured in standard growth medium for the same period. All cell samples were stained with fluorochrome-labeled antibodies specific for CD3, CD4 and CD25. MMC—mitomycin C; Control—cells cultured for 24 h in complete DMEM without *Betonica bulgarica* extract. (**A**) Levels of lymphocytes expressing CD25 in peripheral blood leucocyte samples treated with *Betonica bulgarica* extract, MMC or cultured in standard growth medium and (**B**) T lymphocytes stained for CD4 and CD25 markers.

A main population that showed prominent differences was CD25^+^ lymphocytes. *Betonica bulgarica* extract-treated sample demonstrated markedly elevated level of CD25^+^ lymphocytes (Figure 3A). This result indicated increased level of activated T lymphocytes, which was confirmed by staining for CD3 and CD4 markers. The CD3-negative group did not show significant expression of the CD25 marker. Among the CD3-positive lymphocyte population, the levels of CD4^+^CD25^+^ cells were also markedly higher compared to MMC-treated and untreated control (Figure 3B). These data motivate the assumption that *Betonica bulgarica* extract could activate T cells and/or induce expansion of regulatory T cell populations exerting immunostimulatory and/or immunoregulatory effect. Further experiments are needed to confirm this hypothesis.

**Figure 4:**
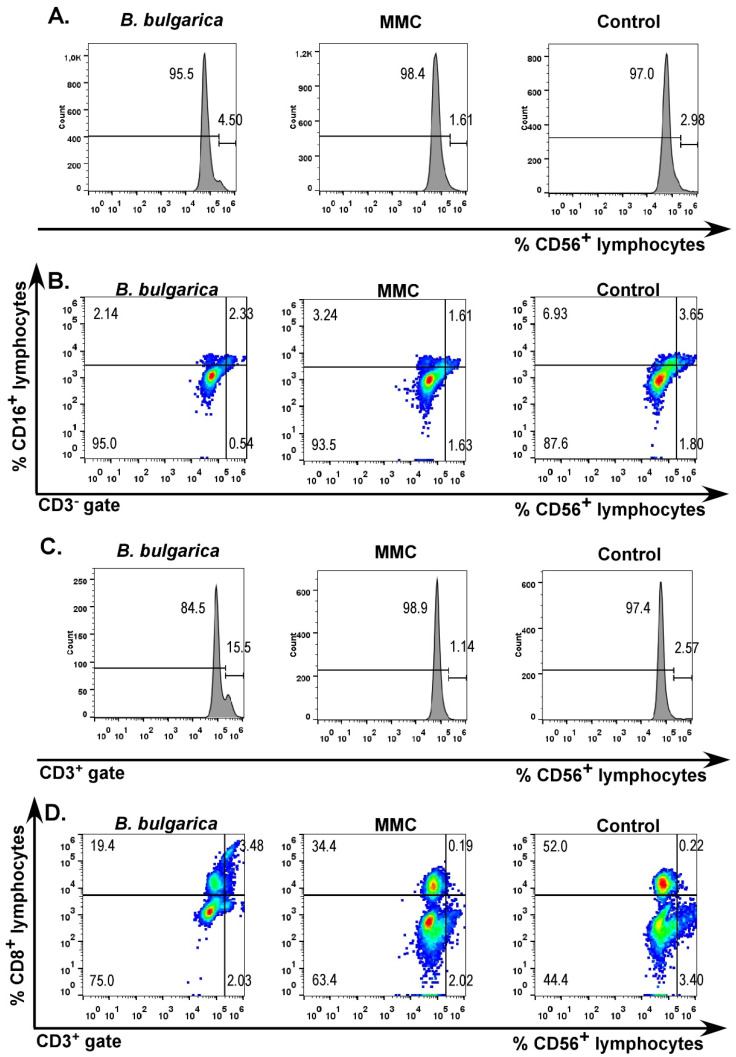
CD56^+^ lymphocyte populations. Flow cytometry evaluations of ex vivo cultured white blood cells for 24 h. Analyses include three types of samples—control cells cultured in standard growth medium, leucocytes treated with 150 μg/mL *Betonica bulgarica* extract and cells cultured in medium containing 100 μg/mL mitomycin C (MMC). (**A**) Histograms representing the total number of CD56^+^ lymphocytes, (**B**) NK cell populations (CD3^−^CD16^+^CD56^+^ lymphocytes), (**C**) CD56^+^ cells from the CD3^+^ population and (**D**) Levels of CD8^+^CD56^+^ in the CD3^+^ group.

Although differences in the levels of main lymphocyte populations were not determined, the analysis of discrete CD56-positive T cells indicated interesting effects. Total levels of CD56^+^ lymphocytes were higher in *Betonica bulgarica* extract-treated sample (Figure 4A). However, analysis of the classical NK cell populations did not show the same tendency and differences between samples treated with extract and controls (Figure 4B). This result pointed out that the increase in CD56-lymphocyte levels is due to modulation in the number of a different lymphocyte group. Interestingly, assessing the level of CD3^+^ lymphocytes, we were able to detect significantly elevated numbers in the *Betonica bulgarica*-treated sample (Figure 4C). 

In addition, when analyzing the CD8^+^ population, we were also able to determine markedly elevated levels of CD56^+^ cells compared to untreated and MMC-treated controls (Figure 4D). These data suggest modulation of cytotoxic lymphocyte populations’ levels induced by treatment with *Betonica bulgarica* extract. A specific regulatory role of these cells could also be assumed based on previous findings [19]. However, additional analyses of cytokines’ expression and cellular interaction effects could reveal the specific mechanism leading to this significant increase in the population of CD3^+^CD56^+^ cells.

## 3. Discussion

*Betonica* species have been used for centuries in folk medicine to treat certain tumors, inflammatory diseases, high blood pressure, ulcers, cough, menopausal problems and as a sedative [11,20,21]. *Betonica bulgarica*, an endemic species that shows significant similarities with the medicinal plant *Betonica officinalis*, has been well characterized in regard to phytochemical and morphoanatomical traits [2,3,4]. Chemical analyses have revealed that *Betonica bulgarica* extracts and essential oils are a rich source of flavonoids and sesquiterpenes with beneficial effects for human health [3,18,20]. However, comprehensive biological activity studies of these samples are needed to determine their potential biomedical use. Although *Betonica bulgarica* is an endemic species, its pharmacological applications cannot be limited because techniques for ex situ conservation have already been developed [22].

The present report is the first to demonstrate an antitumor potential of extract derived from *Betonica bulgarica* inflorescences. Significant cytotoxicity against nontumor human and murine cells was not detected. The reported effect was selective for cervical adenocarcinoma cells because lung adenocarcinoma A549 cells and mouse tumor-derived cells did not show the same level of inhibition as the HeLa cell line. However, a broader antitumor effect of *Betonica bulgarica* could not be excluded and needs to be further investigated with a panel of other tumor cell types with different origins. This hypothesis is supported by previous findings for antiproliferative activity of *Betonica officinalis* against breast and epidermoid carcinoma cells (MCF7 and A431, respectively) in addition to inhibitory effects on HeLa cells [15]. For these analyses, only tumor cell lines were used and cytotoxicity against nontumor cells has not been determined. In comparison, the present research clearly indicates an anticarcinomic specific effect and low cytotoxicity of *Betonica bulgarica* extract against nontumor human and murine cells. Another research group has performed genotoxicity evaluations demonstrating that *Betonica officinalis* extract does not induce chromosome aberrations in normal human lymphocytes in vitro. However, DNA damage was detected and a slight increase in sister chromatid exchange values, which was attributed to the plant extract and potential donor susceptibility [23]. Tumor cells were not included in the study in order to determine stronger genotoxic effect against these type of cells. A report by Ciobanu et al. described effective generation of nanocomposites loaded with polyphenols’ rich fraction obtained from *Betonica officinalis* and demonstrated the ability of *Betonica officinalis* extract to reduce Hep-2 tumor cell viability by interfering with cellular metabolism [24]. However, the reported potential for therapeutic application was not verified by studies on normal nontumor cells. 

The study of Haznagy-Radnai et al. [15] identified iridoids as main extract constituents responsible for the cytotoxic effects of *Stachys* species [15]. In parallel, methanolic extracts obtained from aerial parts of *Betonica bulgarica* populations from different locations have been shown to contain a significant amount of flavonoids, particularly rutin, quercetin and hispidulin [18]. The antitumor properties of these three flavonoids have been proven [25,26,27] as well as their proapoptotic effects [28,29,30]. These findings support the results in the present report and suggest that the detected tumor cell-specific inhibitory activity of *Betonica bulgarica* extract could be based on the main flavonoid compounds (rutin, quercetin and hispidulin). In addition, the present study indicates two major phenolic compounds in *Betonica bulgarica* extracts that could contribute to the determined biological properties. In fact, antitumor properties of chlorogenic acid and caffeic acid have been reported, as well as their potential mechanisms of action in tumor cells [31,32]. Specific inhibitory effects of caffeic acid on cervical adenocarcinoma cells have been described, which supports the present findings and suggests a role of this compound for the selective antitumor effect of *Betonica bulgarica* [33]. Furthermore, a recent report revealed a role of chlorogenic acid for antitumor immunity [34] and highlights a potential link between this phenolic acid and the determined immunomodulatory activity in our studies. However, other natural substances could also contribute to the biological activity of *Betonica bulgarica*. Representative examples are sesquiterpenes, which are abundant in essential oils derived from certain *Betonica* herbs [35]. Notably, antitumor properties have been defined for many sesquiterpenes [36] and particularly for β-caryophyllene, humulene and caryophyllene oxide [37,38] that are the predominant sesquiterpenes in essential oils derived from *Betonica bulgarica* inflorescences [3]. Thus, further detailed studies are needed to define all biologically active substances in *Betonica bulgarica* inflorescence extract and their role for the specific properties of the sample.

The significance of the present work is further emphasized by the results that show alterations in distinct populations involved in different immune responses including antitumor immunity. Hence, in addition to the direct cytotoxic and proapoptotic effect against cervical tumor cells mediated by particular compounds in *Betonica bulgarica* extract, we show that these properties could be supplemented and/or enhanced by induction of specific immune cell types. The most prominent immunophenotypic changes induced by *Betonica bulgarica* were observed on the level of CD25^+^ lymphocytes, as well as on the level of CD56^+^ T cells. In the lymphocytes group, CD25 is expressed by activated cells, natural and inducible regulatory T cells and by memory T cell populations [39,40,41]. Our study shows that a considerable part of the expanded CD25^+^ group following treatment with *Betonica bulgarica* extract belongs to the CD4^+^ subset. This elevated population could be constituted by increased numbers of activated or regulatory T cells. It is possible that this group includes a higher count of both types, either activated or inducible regulatory T lymphocytes—assumptions that can be determined by future experiments and analysis of the expression of additional immune cell markers. Even though the present results could not define the specific type of elevated CD4^+^CD25^+^ T cells, they clearly demonstrate immunophenotypic effect of the extract obtained from *Betonica bulgarica*, which could be immunostimulatory and/or immunoregulatory and may contribute to antitumor and other important biological activities.

Another interesting immunomodulatory effect induced by the extract from *Betonica bulgarica* was related to the group of CD56^+^ lymphocytes. Surprisingly, instead of the classical NK cell population, for which CD56 is an archetypal marker [42], the CD3^+^ lymphocytes showed markedly increased levels of CD56-expressing cells. CD56 has been considered as a phenotypic activation marker that can be inducibly upregulated on the surface of NK cells, T cells, dendritic cells and monocytes [43,44]. In our experimental setting, we were able to detect markedly higher level of CD3^+^CD8^+^CD56^+^ lymphocytes in the sample with peripheral blood leucocytes cultured ex vivo in medium containing *Betonica bulgarica* extract. It has been shown that cells with this phenotype can serve as a potent effector of antitumor immunity after stimulation with cytokines [45,46] and thus, induction of this cell type could contribute to the tumoricidal activity of *Betonica bulgarica* by exerting direct cytotoxic action. In addition, cytolytic CD56^+^ regulatory CD8 lymphocytes have been identified—a subset able to regulate cellular immune responses by killing activated CD4 cells, including pathogenic CD4 lymphocytes [47] and maintaining immune homeostasis [19]. Further experiments are needed to evaluate in detail the properties of the immune cell populations induced by treatment with *Betonica bulgarica* extract, to determine their mechanism of action and the structure–activity relationships and potential beneficial effects on human health.

The two lymphocyte populations that showed marked enhancement after treatment with *Betonica bulgarica* extract can represent important immunomodulatory activity—effector, cytotoxic and regulatory properties. The complex composition of the studied extract could be a main reason for the detected effects on various immune cell populations induced by particular compounds present in the sample or as a result of synergistic action of two or more natural substances. Therefore, a detailed investigation on the composition of the extract and studies on isolated compounds are needed to verify and elucidate these assumptions and validate the biomedical application of *Betonica bulgarica*.

## 4. Conclusions

The present study reports pioneering findings on the biological activity of inflorescence extract obtained from the endemic plant *Betonica bulgarica*. It demonstrates that the extract induces specific antitumor effects against human cervical adenocarcinoma cells but do not exert common cytotoxicity towards nontumor human cells and different murine cell types. The sample from *Betonica bulgarica* showed immunomodulatory activity indicated by elevated levels of CD4^+^CD25^+^ and CD8^+^CD56^+^ lymphocytes in ex vivo extract-treated peripheral blood leucocyte samples. Future experiments could clarify the beneficial functions of these immune cell populations and whether they have activated regulatory and/or cytotoxic phenotype. The present study proves that *Betonica bulgarica* could serve as a potential source of natural compounds for development of improved pharmaceutical products and therapeutic strategies.

## 5. Materials and Methods

### 5.1. Plant Material

*Betonica bulgarica* inflorescences, leaves and stems were collected during the summer of 2020 from naturally growing populations with a defined location (42°45′02″ N, 25°14′18″ E) in Bulgarka Nature Park (central northern part of the Balkan Mountains). The collected material was authenticated by Assoc. Prof. Plamen Stoyanov. Voucher specimens for *Betonica bulgarica* (n. 062646) were deposited at the Herbarium of the University of Agriculture, Plovdiv, Bulgaria. The plant material was dried at room temperature devoid of direct contact to sunlight. The dry inflorescences were mechanically grinded to obtain a powder containing particles smaller than 400 μm. The sample was stored at 16–18 °C in the dark.

### 5.2. Extract Preparation

Ten grams of powdered plant material were mixed with 250 mL 70% methanol aqueous solution and incubated at room temperature in the dark for 24 h. Then, the sample was centrifuged for 10 min at 6000× *g* and the obtained extract was filtered through Whatman No. 1 filter paper (Sigma-Aldrich, Steinheim, Germany). The same procedure was repeated twice and the resulting extracts were pooled. The methanol was removed from the sample via vacuum evaporation at 37 °C using Savant concentrator (SAVANT Instruments Inc., Farmingdale, NY, USA). Five mg dry extract was dissolved in 5 mL 50% DMSO aqueous solution (*w*/*v*) and stored at 4 °C protected from light.

### 5.3. Cell Lines

Six mammalian cell lines were used for evaluation of *Betonica bulgarica* cytotoxicity and antitumor potential: A549 (human lung adenocarcinoma—ATCC^®^ CCL-185™); HeLa (human cervical adenocarcinoma—ATCC^®^ CCL-2™); FL (human amniotic cells—NBIMCC 94); RAW 264.7 (mouse monocyte/macrophage cell line—ATCC^®^ TIB-71™) derived from Abelson murine leukemia virus-induced tumor; NIH/3T3 (mouse embryonic fibroblasts—ATCC^®^ CRL-1658™); LS48 (murine fibroblast-like cell line—DSM ACC 2455) (Biotectid, Germany). Intensive proliferative activity is characteristic for the LS48 cell line and therefore it was used in the experiments to assess potential murine-specific anti-proliferative effects. 

The cells were cultured in complete growth medium consisting of Dulbecco’s modified Eagle’s medium (DMEM) (Sigma-Aldrich Inc., Merck KGaA, Darmstadt, Germany), 10% heat-inactivated fetal bovine serum (FBS) (Sigma-Aldrich Inc., Merck KGaA, Darmstadt, Germany) and an antibiotic antimycotic supplement (100 U/mL penicillin, 100 μg/mL streptomycin and 0.25 μg/mL amphotericin B (Merck KGaA, Darmstadt, Germany). This growth medium is denoted as complete DMEM or standard growth medium in the text. The cells were cultured under sterile conditions in a humidified incubator maintaining 37 °C temperature and 95% atmospheric air/5% CO_2_ content.

### 5.4. In Vitro Cytotoxicity Assays

All cell cultures were expanded in 75 cm^2^ flasks (TPP, Trasadingen, Switzerland). At 80–90% confluency, FL, HeLa, A549, LS48 and NIH/3T3 cells were trypsinized and RAW264.7 cells were mechanically detached using sterile scraper (TPP, Trasadingen, Switzerland). The resulting cell suspensions were diluted to concentration 1 × 10^5^ cells/mL, plated on 96-well plates (TPP, Trasadingen, Switzerland) (200 μL cell suspension/well) and cultured for 24 h under standard conditions. Then, the cells were incubated with plant extract in different concentrations (5, 50, 100, 200 and 250 µg/mL) for 24 h. The extract was diluted in the cell culture medium. All in vitro cytotoxicity assays included control cell samples cultured for 24 h in complete DMEM without *Betonica bulgarica* extract. Cells incubated for 24 h with mitomycin C (Merck KGaA, Darmstadt, Germany) were used as a positive control for cytotoxic effects. Additional control was assayed with the aim to determine the potential inhibitory effect of DMSO that was present in the tested extract—12.5%, 10%, 5%, 2.5%, 0.25% corresponding to DMSO content in 250, 200, 100, 50, 5 µg/mL extract test-sample, respectively. All samples were analyzed in triplicates.

At the end of the 24 h incubation period, 100 μL/well 0.5 mg/mL MTT ([3-(4,5-dimethylthiazol-2-yl)-2,5-diphenyltetrazolium bromide]) (Merck KGaA, Darmstadt, Germany) solution was added to the cell culture plates, which were then incubated for 2 h at 37 °C in darkness. After that, the MTT containing medium was removed and 100 μL/well DMSO (Sigma-Aldrich Inc., Merck KGaA, Darmstadt, Germany) were pipetted to each well. To dissolve the formazan accumulated in viable/metabolically active cells, the plates were incubated for 10–15 min at 37 °C. After that, the absorbance at 570 nm was determined using a Synergy-2 microplate reader (BioTek, Winooski, VT, USA). Percent inhibition of cell vitality and metabolic activity was calculated based on the absorbance data from extract-treated cells and control cells cultured in standard conditions. Inhibition levels detected for DMSO controls were subtracted from the values of the corresponding samples.

### 5.5. Isolation of Leucocytes and Ex Vivo Treatment

Peripheral blood was collected in BD Vacutainer^®^ K2EDTA tubes (Becton, Dickinson and Company (BD), Franklin Lakes, NJ, USA) from the cubital vein of healthy volunteers (n = 3, 27–35 years of age). Standard complete blood count parameters of the obtained samples were analyzed and determined to be in the normal range. The blood was centrifuged at 1500 rpm/min for 20 min at room temperature. The plasma was discarded, which was followed by lysis of erythrocytes with 0.84% NH_4_Cl buffer. Then, the samples were washed twice with 10 mL Dulbecco’s modified phosphate-buffered saline (DPBS) (Merck KGaA, Darmstadt, Germany). The isolated leukocytes were pooled and centrifuged at 1000 rpm/min for 10 min. Then, the cells were resuspended in complete growth medium and seeded on 6-well culture plates (TPP, Trasadingen, Switzerland) for 24 h ex vivo treatment with *Betonica bulgarica* extract. The test-sample was diluted in the cell culture medium to a final concentration of 150 μg/mL. The experiment included control leucocytes cultured in complete DMEM without extract and a negative control treated with 100 μg/mL mitomycin C (MMC). The cells were grown in a humidified incubator at 37 °C and 5% CO_2_. After 24 h, the treated and control leucocytes were harvested and centrifuged at 1000 rpm/min for 10 min. The cell pellets were resuspended in FACS buffer (DPBS, supplemented with 5% fetal bovine serum and 0.05% NaN_3_).

### 5.6. Flow Cytometry Analyses

Peripheral blood leucocytes were cultured ex vivo for 24 h in standard growth medium containing *Betonica bulgarica* extract or mitomycin C. Effects on different immune cell populations were determined in comparison to cells cultured for the same test-period in complete DMEM without extract or MMC. For immunophenotypic analyses, control, MMC-treated and extract-treated leucocytes were stained with fluorochrome-labeled antibodies specific for CD3, CD4, CD8, CD19, CD25 and CD56 markers (BD Pharmingen™, BD Biosciences). The antibodies were added to the cell samples according to the manufacturers’ recommendation and incubated in the dark for 15 min at room temperature. Then, the cells were washed twice with FACS buffer and analyzed on a Cytomics FC500 flow cytometer (Beckman Coulter, Pasadena, CA, USA).

To determine whether exposure to *Betonica bulgarica* extract could induce apoptosis, experiments with HeLa cells and ex vivo isolated peripheral blood leucocytes were performed. Following 24 h culture in medium containing 150 μg/mL extract, the cells were stained with fluorochrome-conjugated Annexin V and subjected to flow cytometry analysis. This experimental setup allowed determination of potential tumor-specific or common proapoptotic effect. For this aim, 1 × 10^6^ cells/mL (HeLa or peripheral blood leucocytes) were seeded on 6-well plates (TPP, Trasadingen, Switzerland) and cultured for 24 h in complete DMEM medium containing 150 μg/mL *Betonica bulgarica* extract. Cells treated with 100 μg/mL mitomycin C for the same time-period served as a positive control for the assay. Untreated control (cells cultured for 24 h under standard conditions in complete DMEM) was also analyzed. All samples were assayed in triplicates. After 24 h, the cells were harvested, centrifuged and resuspended in 500 μL binding buffer. Each sample was stained with 5 μL Annexin V-FITC (Abcam, Cambridge, UK) for 20 min at room temperature in the dark. Then, the cells were washed and apoptotic cell levels were analyzed by flow cytometry using a Cytomics FC 500 flow cytometer (Beckman Coulter, Pasadena, CA, USA).

### 5.7. Analyses of Phenolic Content

Total phenolic content was determined as previously described by Mamelona et al. using the Folin–Ciocalteu reagent [48]. The samples were analyzed spectrophotometrically at 760 nm and the results were presented as mg gallic acid equivalent (GAE) per 100 g dry weight (DW) sample (mg GAE/100 g). All samples were reported in triplicates.

Major phenolic compounds were determined by high-performance liquid chromatography (HPLC) analysis applying the method reported by Katsarova [49]. HPLC system (Varian Inc., Agilent, Walnut Creek, CA, USA) equipped with ProStar 230 solvent delivery module and Hitachi C18 AQ (250 mm × 4.6 mm, 5 μm) column was used. The system of solvents included deionized water adjusted to pH 3.7 with phosphorus acid and acetonitrile in gradient state. Star Chromatography Workstation software (version 6.30) (Agilent, Walnut Creek, CA, USA) was used for the analyses. Chlorogenic acid and caffeic acid were detected at 360 nm. Results were calculated with standard curves for each analytical standard and expressed as mg/100 g DW.

### 5.8. Statistics

Statistical analyses were performed using the StatView software (version 5.0) (SAS Institute, Carry, NC, USA). Analysis of variance (ANOVA) was applied to determine differences between test-groups. Significant ANOVA result analysis was followed by Fisher’s protected least significant difference (PLSD) test for pairwise comparison of experimental groups. Finally, the non-parametric Mann–Whitney U-test was applied to confirm statistically significant differences between the median of two groups of data, i.e., the control cells and *Betonica bulgarica*-treated cells. *p* values lower than 0.05 were regarded as statistically significant.

## Figures and Tables

**Table 1:** In vitro cytotoxicity of *Betonica bulgarica* extracts obtained from different aerial parts of the plant.

*B. bulgarica* Extracts	Parameters	HeLa	A549	FL	NIH/3T3	LS48	RAW264.7
Leaves	% inhibition	55.7	2.1	52.6	26.3	25.7	29.3
	IC_50_ (μg/mL)	132.95	-	147.2	-	-	-
Stem	% inhibition	41.9	4.4	6.9	40.8	4.3	-
	IC_50_ (μg/mL)	231.34	-	-	194.5	-	-
Inflorescence	% inhibition	66.3	15.9	3.3	30.8	11	30.4
	IC_50_ (μg/mL)	119.2	-	-	-	-	-

Data represent mean values. % inhibition of cellular viability and metabolic activity has been determined after 24 h treatment with 150 μg/mL *B. bulgarica* extract. (-) not determined (no inhibition).

**Table 2:** Evaluations of phenolic compounds in *Betonica bulgarica* extracts.

Samples	Total Phenolic Content (mg GAE/100 g)	Major Phenolic Compounds (mg/100 g)
*Betonica bulgarica* (leaves)	4651.9 ± 304	Chlorogenic acid: 4.37 ± 0.39 Caffeic acid: 0.20 ± 0.01
*Betonica bulgarica* (stem)	2779.3 ± 51.1	Chlorogenic acid: 3.72 ± 0.12 Caffeic acid: 0.10 ± 0.02
*Betonica bulgarica* (inflorescence)	3177.8 ± 56.5	Chlorogenic acid: 4.25 ± 0.73 Caffeic acid: 0.20 ± 0.1

## Data Availability

Data are contained within the article or available from the corresponding author upon request.

## Abbreviations

ACC	animal cell culture
ANOVA	analysis of variance
ATCC	American Type Culture Collection
CD	cluster of differentiation
DMEM	Dulbecco’s modified Eagle medium
DMSO	dimethyl sulfoxide
DPBS	Dulbecco’s modified phosphate-buffered saline
DSM	German Collection of Microorganisms and Cell Cultures
DW	dry weight
EDTA	ethylenediaminetetraacetic acid
FACS	fluorescence-activated cell sorting
FBS	fetal bovine serum
FITC	fluorescein isothiocyanate
GAE	gallic acid equivalent
HPLC	high-performance liquid chromatography
MMC	mitomycin C
MTT	[3-(4,5-dimethylthiazol-2-yl)-2,5-diphenyltetrazolium bromide]
NBIMCC	Bulgarian National Bank for Industrial Microorganisms and Cell Cultures
NIH	National Institutes of Health
PLSD	protected least significant difference
